# Assessment of the Peripheral and Central Auditory System in Infants Whose Mothers Tested Positive for COVID-19 During Pregnancy

**DOI:** 10.3390/children11121527

**Published:** 2024-12-16

**Authors:** Jheniffer Queiroz Raimundo, Milaine Dominici Sanfins, Piotr Henryk Skarzynski, Magdalena Beata Skarżyńska, Maria Francisca Colella-Santos

**Affiliations:** 1 School of Medical Sciences, State University of Campinas, Campinas 13083-887, Brazil; j199534@dac.unicamp.br (J.Q.R.); mfcolell@unicamp.br (M.F.C.-S.); 2 Department of Speech-Hearing-Language, Universidade Federal de São Paulo, São Paulo 04044-020, Brazil; 3 Post-Graduate Program in Clinical Audiology, Instituto de Ensino e Pesquisa Albert Einstein, São Paulo 05652-000, Brazil; 4 Department of Teleaudiology and Screening, World Hearing Center, Institute of Physiology and Pathology of Hearing, 05-830 Kajetany, Poland; p.skarzynski@csim.pl; 5 ENT Department, Maria Curie-Skłodowska University, 20-031 Lublin, Poland; 6 Center of Hearing and Speech Medincus, 05-830 Kajetany, Poland; 7 Department of Otolaryngology, Institute of Sensory Organs, 05-830 Warsaw, Poland; 8 Heart Failure and Cardiac Rehabilitation Department, Medical University of Warsaw, 02-091 Warsaw, Poland; 9 World Hearing Center, 05-830 Kajetany, Poland; 10 Department of Pharmacotherapy and Pharmaceutical Care, Medical University of Warsaw, 02-091 Warsaw, Poland; m.skarzynska@csim.pl; 11 Institute of Sensory Organs, 05-830 Kajetany, Poland; 12 Center of Hearing and Speech, 05-830 Nadarzyn, Poland

**Keywords:** COVID-19, hearing, infants, SARS-CoV-2

## Abstract

Introduction: Coronavirus disease 2019 (COVID-19) is an infectious disease caused by the Severe Acute Respiratory Syndrome Coronavirus 2 (SARS-CoV-2), a new member of the coronavirus family. While respiratory transmission is the main route, concerns have arisen regarding possible vertical transmission, which refers to the transmission of the virus from mother to fetus through the dissemination of viral particles in the amniotic fluid. Fetal viral infection via the placenta can affect the formation of the auditory system and lead to congenital hearing disorders. The aim of this research was to investigate the effects of vertical exposure to COVID-19 on the auditory system of newborns. Methodology: The study included a study group (SG) and a control group (CG). Selected during neonatal hearing screening, the SG consisted of 23 infants up to 1 year old whose mothers had been infected with SARS-CoV-2. The CG consisted of 15 infants whose mothers became pregnant after the end of the pandemic, had tested negative for COVID-19, and had no respiratory symptoms during pregnancy. The assessments for both groups were brainstem auditory evoked potentials (BAEPs), tympanometry, transient evoked otoacoustic emissions (TEOAEs), and distortion product otoacoustic emissions (DPOAEs). The research was divided into two studies, one cross-sectional and the other longitudinal. Results: All participants exhibited absolute latencies within the normal range for waves I, III, and V, although in the SG, there was a statistically significant increase in the latency of wave I in the left ear. In terms of OAEs, in the SG, there appeared to be a tendency for TEOAEs to be absent at high frequencies. Over several months, there was a general decrease in the amplitude of high-frequency responses in both TEOAEs and DPOAEs. Conclusion: No evidence was found that vertical exposure to COVID-19 causes hearing loss, although there were signs of possible deterioration in hair cell functioning.

## 1. Introduction

Coronavirus disease 2019 (COVID-19), caused by the Severe Acute Respiratory Syndrome Coronavirus 2 (SARS-CoV-2) virus, was initially reported in Wuhan, Hubei Province, China, and spread globally, affecting more than 120 countries, becoming a worldwide public health concern [1]. COVID-19 transmission occurs predominantly via the respiratory route, although studies have presented evidence of the possibility of vertical transmission, in which the virus is transferred from the mother to the fetus through viral particles in the amniotic fluid, hematogenous transplacental transmission, exposure to contaminated maternal blood and secretions, or during breastfeeding [2].

Viral infections during pregnancy, such as measles, rubella, toxoplasmosis, herpes, cytomegalovirus, HIV, and Zika virus, have been associated with alterations in the formation of the auditory system and congenital malformations in newborns, representing risk factors for hearing loss, as defined by the Joint Committee on Infant Hearing [3].

One entry point for the SARS-CoV-2 virus into the auditory system is through receptors for angiotensin-converting enzyme 2 and transmembrane serine protease 2, present in the epithelium of the middle and inner ear, which may justify findings of alterations in otoacoustic emissions (OAEs) that point to lesions at the hair cell level [4].

The literature indicates that viral pathogens can cause damage to the peripheral and central auditory systems, leading to permanent or temporary alterations in auditory function. Hearing loss is one of the alterations resulting from viral infections, which can be unilateral or bilateral, ranging from mild to profound, and predominantly sensorineural, but does not exclude the possibility of conductive and mixed losses [5,6].

Despite the knowledge about viral infections and possible alterations in the central and peripheral auditory systems, the effects of COVID-19 in infants exposed to the SARS-CoV-2 virus through vertical transmission are still unknown, and studies on the subject are limited. There is a lack of prospective studies that have looked at possible impacts through longitudinal follow-ups. Considering the limited number of studies on the impacts caused by SARS-CoV-2 infection on the auditory system and auditory development of newborns whose mothers tested positive for COVID-19 during pregnancy, research on the subject can help give a deeper understanding of the possible auditory impacts caused by vertical transmission of SARS-CoV-2 infection. This study also seeks to better understand the possible outcomes of these cases, aiming to establish appropriate approaches to the diagnosis of auditory alterations and/or delays in the development of auditory skills.

The present study aimed to analyze the effects of COVID-19 on the auditory system of newborns whose mothers tested positive for the SARS-CoV-2 virus during pregnancy and looked at the possible impacts on the peripheral and central auditory systems up to 12 months after birth.

## 2. Materials and Methods

### 2.1. Statement of Ethics

This is a primary observational study, both cross-sectional and longitudinal, approved by the institution’s Research Ethics Committee (Approval No. 5.454.075 and CAAE 56309121.7.0000.5404).

### 2.2. Participants

Infants aged 1 to 12 months with Type A tympanograms, and whose mothers had COVID-19 during the gestational period (confirmed by RT-PCR test or rapid antigen test), were included in a study group (SG). In a control group (CG), we included infants of the same age range as the SG, with Type A tympanograms, and who were born after the World Health Organization’s declaration of the end of the pandemic. Mothers in the CG had never tested positive for COVID-19 and had no history of respiratory symptoms during pregnancy. The exclusion criteria for both the SG and CG included the presence of risk indicators for hearing loss (RIHL). Mothers in both groups were tested for infectious diseases during pregnancy, and any children exhibiting risk indicators for hearing loss, as defined by the Joint Committee on Infant Hearing (JCIH), were excluded from the study.

To recruit participants for the SG, a preliminary survey was conducted by a multidisciplinary team at a tertiary hospital to identify pregnant women infected with the SARS-CoV-2 virus. These women were contacted by phone to bring their newborns to the audiology laboratory of the same institution at a scheduled date and time.

The guardians received an Informed Consent Form (ICF) and were invited to complete a questionnaire regarding the gestational period, information about the birth, and the newborns’ first months of life. This research was divided into two studies: one cross-sectional and the other longitudinal. The first study (Study I) aimed to verify whether COVID-19 during the gestational period had impacted the peripheral and central auditory systems of newborns. For this purpose, a single assessment was conducted when the child was between 1 and 12 months old, and the following procedures were performed: tympanometry, transient evoked otoacoustic emissions (TEOAEs), distortion product otoacoustic emissions (DPOAEs), and brainstem auditory evoked potentials (BAEPs).

Mothers of children who had participated in the cross-sectional study and whose children were aged between 1 and 3 months were invited to participate in a separate longitudinal study (Study II), in which a follow-up assessment was performed between 7 and 12 months of age. There were only 7 participants in Study II, which meant that statistical significance was hard to establish.

### 2.3. Procedures

Tympanometry was performed to verify aspects related to the middle ear and produce tympanogram curves. The following parameters were considered for normal middle ear function: Type A tympanograms with peak compliance between 0.3 and 1.3 mL and pressure between −100 and +100 dPa [7]. The equipment used for this procedure was the Titan from Interacoustics. The probe tone used depended on the child’s age:

- Newborn to 3 months: 1000 Hz probe tone;
- 3 to 9 months: 1000 Hz probe tone, and in case of abnormal results, a second evaluation using a 226 Hz tone;
- Infants older than 9 months: 226 Hz probe tone.

Transient evoked otoacoustic emissions (TEOAEs) were assessed at frequencies of 1, 2, 3, 4, and 5 kHz. For distortion product otoacoustic emissions (DPOAEs), the frequencies of 0.5, 1, 1.5, 2, 3, 4, 5, 6, 7, 8, 9, and 10 kHz were evaluated. In both procedures, the minimum values considered were 98% stability and >90% reproducibility, as described by Dhar and Hall [8]. For TEOAEs and DPOAEs, a signal-to-noise ratio of ≥6 dB was considered “present”, and <6 dB was considered “absent” for each frequency [9,10]. The equipment used for electroacoustic evaluation was the Audio-Smart from Neurosoft.

For electrophysiological evaluation, the same Audio-Smart equipment was used. For BAEPs, a click stimulus at an intensity of 80 dBnHL was used for integrity analysis. The parameters adopted were a duration of 40 µs, 2000 sweeps, rarefaction polarity, a window of 10/15 milliseconds, and a bandpass filter of 0.15–3 kHz. Electrode placement followed the international 10/20 system, with the reference electrode at Fpz, a ground electrode used, and inverted electrodes on the mastoid processes (M2, right; M1, left). Impedance had to be less than 3 kΩ.

The analysis included absolute latency values (ms) of waves I, III, and V, the amplitude (µV) of waves I and V, the interpeak intervals (ms) of I–III, III–V, and I–V, and the amplitude ratio V/I. The marking was performed by two researchers and checked by a third, based on the normality criteria proposed by Gorga [10].

### 2.4. Data Analysis

For statistical analysis, a Mann–Whitney test with continuity correction was used to compare the control group with the study group. For the analysis of Study II, the longitudinal study, which involved paired data (when the same subject served as both study and control), cases with no responses at either time point were excluded. This exclusion (only for this analysis) was conducted variable by variable using the Wilcoxon test.

To control for the Type I error rate due to the multiple statistical tests performed, the Bonferroni correction was applied. Given that 18 statistical tests were conducted, the *p*-value threshold for statistical significance was adjusted to 0.0028. After applying this correction, the *p*-values were recalculated, and any previously significant results were reevaluated.

The alpha significance level was set at 0.05, and *p*-values <0.05 are highlighted in bold in the tables. R software version 4.4.0 was used to apply statistical methods and create graphs. The effect size was calculated using G*Power software version 3.1.9.2.

## 3. Results

**Figure 1:**
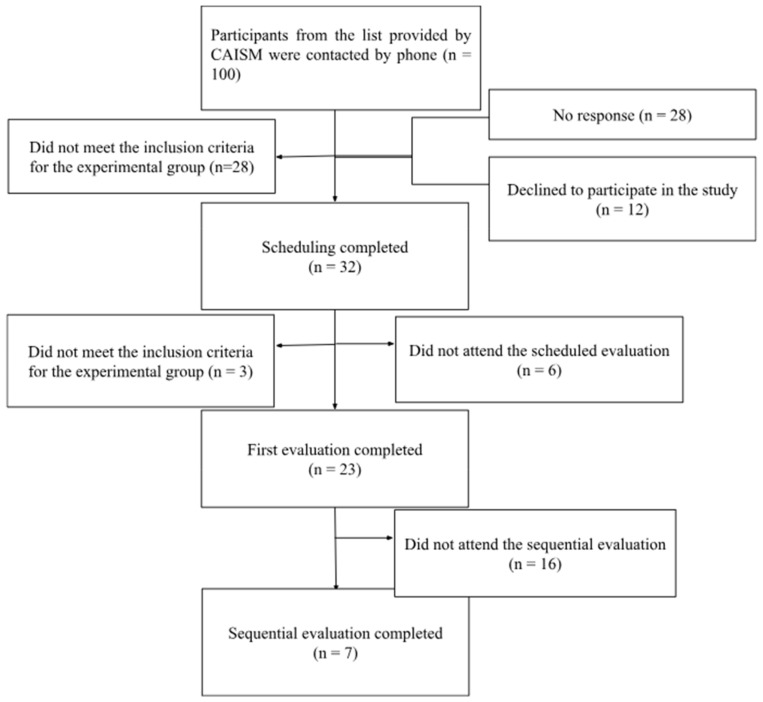
Description of the call and stages of the study.

Figure 1 describes the steps for recruiting the EG, providing data on the number of participants contacted, how many did not meet the inclusion criteria for the EG, how many did not attend the scheduled appointments, and how many declined to participate in the study.

To facilitate the presentation of the results, this section is divided into two parts: first, the results of Study I (cross-sectional) are presented, followed by the results of Study II (longitudinal).

### 3.1. Study I (Cross-Sectional)

The control group consisted of 15 participants aged 1 to 12 months. The age distribution is described in Table 1, with 7 (46.6%) female and 8 (53.3%) male participants. The study group consisted of 23 participants (Table 1), aged 1 to 12 months (mean = 7.0; SD = 4.0), with 11 (47.8%) female and 12 (52.2%) male participants.

Regarding the data related to the gestational period, two (9.5%) mothers reported the need for hospitalization, eighteen (85.7%) experienced symptoms during the acute phase of the disease, and one (4.8%) was asymptomatic. It is important to note that the number of pregnant women was lower than the total number of participants in the study due to a twin pregnancy.

Concerning the gestational trimester in which the mother tested positive for the SARS-CoV-2 virus, four (17.4%) tested positive in the first trimester, fourteen (60.9%) in the second trimester, and five (21.7%) in the third trimester.

**Figure 2:**
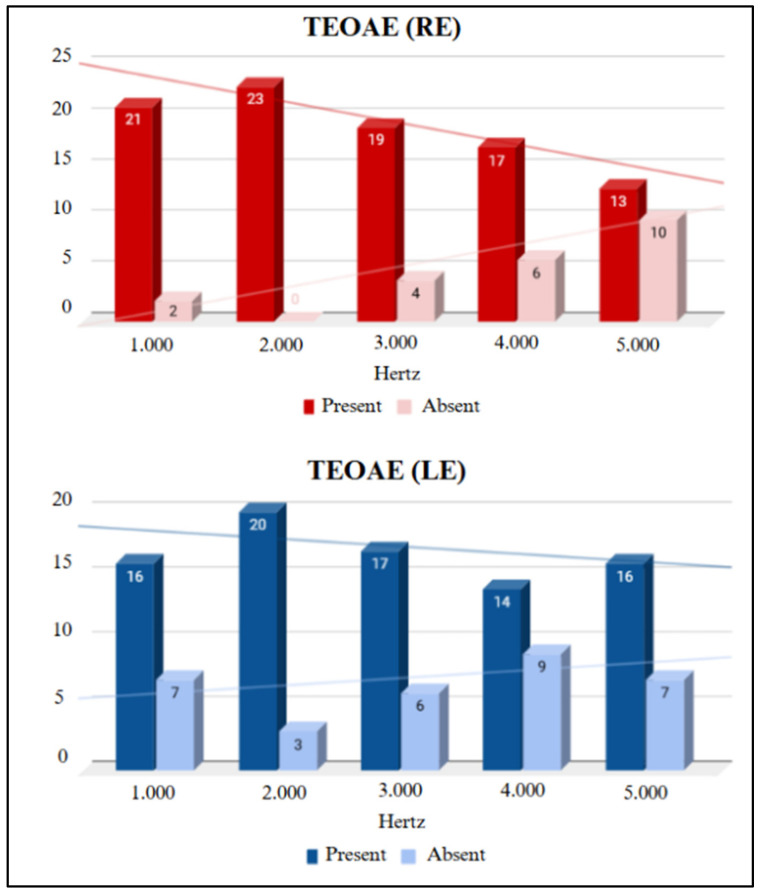
Presence and absence of TEOAEs by frequency in the study group (SG). Key: RE, right ear; LE, left ear.

**Figure 3:**
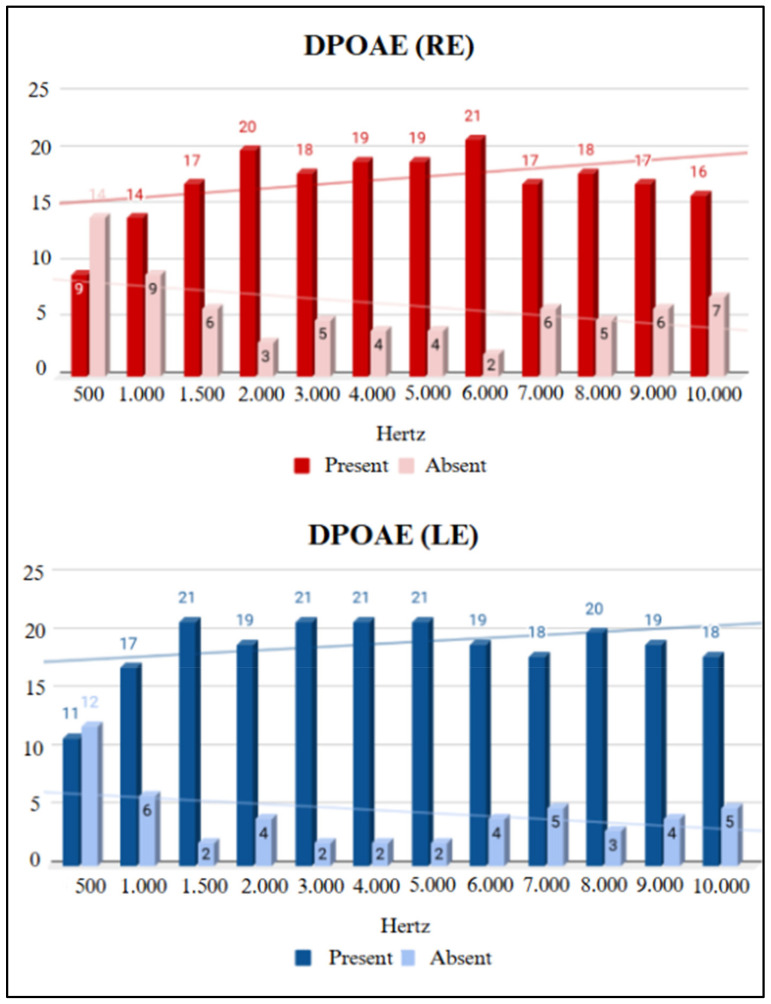
Presence and absence of DPOAEs by frequency in the study group (SG). Key: RE, right ear; LE, left ear.

All participants showed a Type A tympanogram curve. Figure 2 presents the results for the presence and absence of TEOAEs by frequency, and Figure 3 shows the results for DPOAEs.

Regarding the electrophysiological evaluation through the Brainstem Auditory Evoked Potential (BAEPs), the absolute latency values (ms) of waves I, III, and V, considering the study group and the control group, are described in Table 2. The results of the I–III, III–V, and I–V interpeak intervals are depicted in Table 3, and the amplitude values (µV) of waves I and V and the V/I ratio are shown in Table 4. All participants demonstrated reproducibility in the waveforms.

### 3.2. Study II (Longitudinal)

The study group (SG) consisted of seven participants. Regarding the gestational period in which the mother tested positive for the SARS-CoV-2 virus, one (14%) was in the first trimester, four (57%) in the second trimester, and two (29%) in the third trimester. All participants presented type A tympanometric curves.

**Figure 4:**
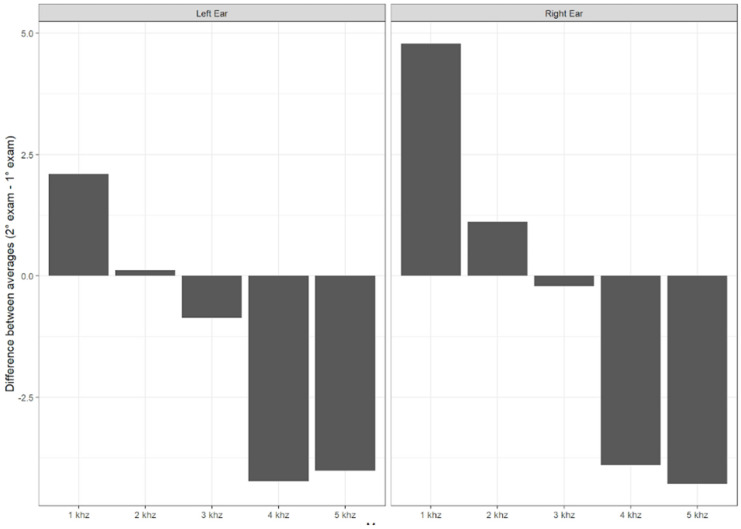
Average difference between the response amplitudes from the initial evaluation and the second evaluation of TEOAEs.

**Figure 5:**
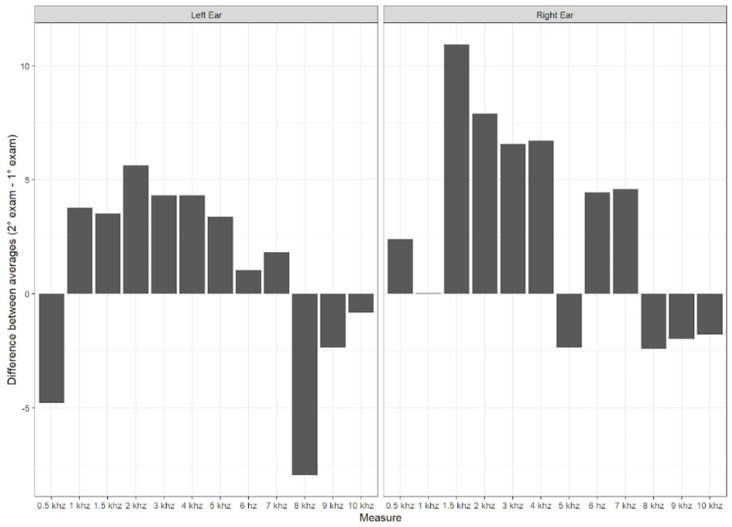
Average difference between the response amplitudes from the initial evaluation and the second evaluation of DPOAEs.

Concerning the electroacoustic evaluation in the longitudinal study, the amplitude parameter was initially analyzed by ear and gender, and statistically significant differences were observed between the ears. As a result, the subsequent analyses were performed, considering each side separately. Figure 4 shows the average difference between the amplitudes of the initial evaluation and the second evaluation of the TEOAEs, and the same is shown in Figure 5 for the DPOAE evaluation.

**Figure 6:**
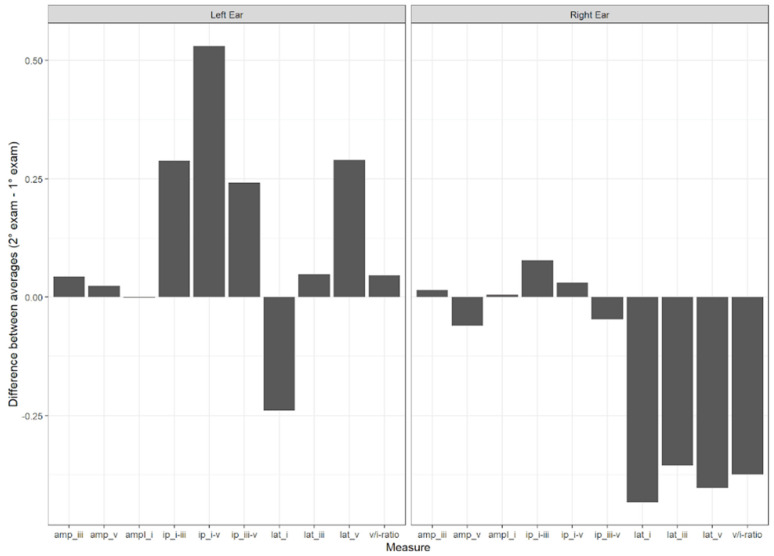
Average difference between the measures (amplitude and absolute latency of waves I, III, and V, interpeak intervals I–III, III–V, and I–V, and amplitude ratio between wave V and wave I) from the initial evaluation and the second evaluation of the BAEPs. Key: amp = amplitude; Ip = interpeak interval; lat = latency.

Figure 6 shows the average difference between the measures from the initial evaluation and the second evaluation of the BAEPs.

## 4. Discussion

Even after applying statistical corrections, the results did not provide sufficient evidence to reject the null hypothesis, likely due to the limitations imposed by the sample size. Nonetheless, this study represents a valuable starting point, emphasizing the necessity of further research with larger sample sizes to enable more robust and reliable conclusions. It is important to note that the minimum detectable effect in this study demonstrated a low statistical power, reinforcing the impact of the sample size constraint.

The findings presented here emphasize the importance of continuing research in this area, as they open avenues for future studies that may provide more conclusive evidence regarding the impact of the SARS-CoV-2 virus on the peripheral and central auditory system of newborns is still unknown, especially regarding long-term effects.

Most studies are retrospective and focus on screening procedures, with no prospective studies, including diagnostic tests or longitudinal studies. This study is the first prospective, cross-sectional, and longitudinal study using the diagnostic of BAEPs (Brainstem Auditory Evoked Potentials).

One limitation in better understanding the impacts of COVID-19 on infants is the variation that occurs in the auditory pathways due to maturation. This research did not find hearing loss in any of the evaluated infants; however, it raised some electroacoustic and electrophysiological findings that require further investigation and more extensive research.

Regarding the electroacoustic evaluation, a trend was observed where the number of absent TEOAE responses increased at higher frequencies. The absence of responses at high frequencies raises the possibility of damage to cochlear hair cells. In the longitudinal study, a lower amplitude of TEOAE responses at frequencies of 4 and 5 kHz was noted when comparing the first evaluation with the second, and the same occurred with DPOAEs at 8, 9, and 10 kHz. This finding of more absent responses at high frequencies in otoacoustic emissions was found in individuals exposed to pathogens that damage cochlear structures, such as in cases of fetal viral infections [11]. Therefore, long-term studies are essential to verify whether SARS-CoV-2 infection behaves similarly to other fetal viral infections.

A study by Celik et al. [6] evaluated 36 infants whose mothers were diagnosed with COVID-19 during pregnancy (experimental group) using TEOAEs and DPOAEs. The results indicated a statistically significant difference between the experimental group and the control group (with no history of COVID-19), with lower TEOAE amplitudes in the experimental group at frequencies of 3 and 4 kHz compared to the control group. Seven other studies have also evaluated newborns whose mothers tested positive for SARS-CoV-2 during the gestational period using a Neonatal Hearing Screening protocol involving TEOAEs [12,13,14,15,16,17,18]. All of these studies presented TEOAE results in the screening without statistically significant differences in the study group. A study on an adult population exposed to COVID-19 showed a lower average amplitude response in the transient-evoked otoacoustic emissions test when compared to the control group, suggesting impairment in the functioning of cochlear hair cells [19].

Regarding the central auditory system, the Automated Brainstem Auditory Evoked Potential (A-BAEP) has been used in studies to investigate the effects of COVID-19 on the brainstem. In the present study, the absolute latency of waves I, III, and V were within the normal range for all participants (according to the normality values proposed by Gorga [10]). However, elevated values for the study group compared to the control group in the latency of wave I in the left ear (Table 1). Regarding wave V in the left ear, although the *p*-value (0.051) between the control group and the study group did not reach statistical significance, this result is likely due to the sample size, not excluding the possibility of significant findings with a larger sample. For the interpeak latencies (I–III, III–V, and I–V), differences between the groups were observed in the III–V and I–V intervals for the left ear, with the study group exhibiting longer latencies. The analysis of BAEP amplitude reflects the magnitude of the electrical response and was affected by the number of nerve fibers involved. The results did not reveal a statistically significant difference in the amplitudes of waves I and V. Regarding the V/I ratio (the relationship between the amplitude of wave I and wave V), no statistically significant differences were found between the groups.

In the longitudinal study, it was noted that in the right ear, there was a decrease in the absolute latency values of waves I, III, and V, findings that are expected according to the literature due to maturation aspects. It is known that the central auditory system matures from the first months of life up to around 18 months, when these values become similar to those of adults. Hall [20] reports that with age, latency values decrease. However, the same does not occur for waves III and V in the left ear, where these latency values increased over time. Although the statistics do not indicate these differences as significant, this indicates that COVID-19 may have an impact on the auditory system over time, so more longitudinal research is needed.

Other studies that have used the A-BAEP test to investigate changes caused by gestational COVID-19 infection, all conducted during the screening phase, have concluded that coronavirus infection during pregnancy is not a risk factor for hearing loss [21,22,23,24,25,26,27]. The study by Alan et al. [21] found that neonates whose mothers tested positive for SARS-CoV-2 during pregnancy were more likely to present a “fail” result in the first A-BAEP test during neonatal hearing screening compared to the control group. However, our retest results involving A-BAEP did not differ significantly between the groups, so we are unable to comment on this aspect.

Literature reviews [28,29] highlight that there is an asymmetry between the ears, showing an advantage of the right ear over the left, which is evident in infants using BAEPs. The difference was also found in this research. Despite the statistically significant difference between the ears tested for latency and amplitude values, these findings are not considered clinically relevant because this population is in the process of auditory pathway maturation.

This research did not find cases of late-onset hearing loss, similar to the study by Apa et al. [13], which followed for one year the children born to mothers who had contracted COVID-19. In this study, there were no cases of moderate or severe bilateral hearing loss during this period; however, the presence of findings compatible with middle ear dysfunction was observed.

It is known that viral agents can damage both the cochlea and the brainstem. This occurs because some viruses cause direct lesions in the stria vascularis, spiral ganglion, and even the organ of Corti, resulting from the immune mechanism of the infected person as a response to the proteins produced by the virus and its entry point for secondary bacterial infections [30,31]. It is noteworthy that due to the variability of COVID-19 symptoms, its effect is still uncertain in some organs, such as the cochlea.

The integrity of the auditory pathways is fundamental for the maturation of the auditory system and is essential for the overall development of the infant, particularly in social, cognitive, and language aspects. The study by Edlow et al. [32] sought to investigate the impacts on neurodevelopment of infants resulting from exposure to SARS-CoV-2 during the gestational period and found a higher risk of adverse neurodevelopmental outcomes during the first year of life among offspring exposed to SARS-CoV-2. This work highlights the importance of studies that follow the auditory and global development of children exposed to COVID-19 through vertical transmission.

Given the findings of this research and the review of studies on the impacts of vertical exposure to the COVID-19 virus on infants, it is evident that there is a need for studies evaluating the integrity of the auditory pathways by monitoring beyond the neonatal hearing screening stage. Furthermore, there is a lack of longitudinal studies evaluating the potential long-term impacts of the SARS-CoV-2 virus.

We conclude that it is crucial to conduct prenatal follow-ups to investigate symptoms related to COVID-19 during pregnancy. It is also important to subsequently monitor newborns to identify potential lesions in the peripheral and central auditory systems resulting from exposure to SARS-CoV-2 during gestation. The aim is to achieve early audiological diagnosis, which can reduce impacts on general child development.

## 5. Conclusions

This study found no evidence that vertical exposure to the SARS-CoV-2 virus in infants is related to cases of hearing loss. However, statistically significant differences were identified between the control group and the study group. Despite the absence of cases of hearing loss, there was a prevalence of absent responses at high frequencies in otoacoustic emissions, leading to the possibility that there had been damage to hair cells. Moreover, a decrease in otoacoustic emissions amplitude over time and with increasing frequency was observed.

## Figures and Tables

**Table 1:** Sample distribution by sex and age group.

Age (Months)	Control Group, CG		Study Group, SG		Total
	M	F	M	F	
0 to 3	3	5	3	6	17
3 to 6	3	1	1	1	6
6 to 8	1	1	5	2	9
≥9	1	0	3	2	6
Total	8	7	12	11	38

Key: M = male; F = female.

**Table 2:** Absolute latency values (ms) of waves I, III, and V in the Auditory Brainstem Response for both the study group and the control group.

	Ear	Group	*N*	Mean	Median	SD	Q1	Q3	Min	Max	*p*-Value
I	RE	Control	15	1.75	1.59	0.38	1.48	1.93	1.42	2.59	0.11678
	RE	Study	23	1.94	1.72	0.60	1.55	2.11	1.30	3.92	
	LE	Control	15	1.71	1.59	0.31	1.53	1.71	1.43	2.33	0.01394 *
	LE	Study	23	2.00	1.91	0.40	1.70	2.30	1.43	3.04	
III	RE	Control	15	4.50	4.60	0.51	4.16	4.85	3.30	5.13	0.86500
	RE	Study	23	4.50	4.37	0.55	4.19	4.74	3.55	6.14	
	LE	Control	15	4.35	4.33	0.56	3.99	4.82	3.23	5.03	0.67719
	LE	Study	23	4.49	4.50	0.46	4.22	4.84	3.49	5.13	
V	RE	Control	15	6.49	6.43	0.56	6.27	6.80	5.10	7.51	0.95478
	RE	Study	23	6.41	6.40	0.63	6.27	6.72	5.18	7.62	
	LE	Control	15	6.39	6.40	0.54	6.15	6.83	5.18	7.25	0.05157
	LE	Study	23	6.65	6.59	0.56	6.47	6.92	5.10	7.46	

Key: Q1, first quartile; Q3, third quartile; N, number of participants; OD, right ear; OE, left ear; bold numbers, *p* ≤ 0.05 *.

**Table 3:** Results of interpeak intervals I–III, III–V, and I–V in the Auditory Brainstem Response for both the study group and the control group.

	Ear	Group	*N*	Mean	Median	SD	Q1	Q3	Min	Max	*p*-Value
I–III	RE	Control	15	2.77	2.81	0.63	2.52	3.14	1.10	3.63	0.08209
	RE	Study	23	2.56	2.59	0.23	2.39	2.70	2.02	3.07	
	LE	Control	15	2.64	2.58	0.66	2.33	3.23	1.21	3.50	0.33526
	LE	Study	23	2.49	2.60	0.29	2.33	2.72	1.85	2.80	
III–V	RE	Control	15	1.98	1.96	0.38	1.80	2.16	1.28	2.75	0.76241
	RE	Study	23	1.91	1.95	0.38	1.69	2.12	1.07	2.78	
	LE	Control	15	2.30	2.26	0.36	2.12	2.46	1.80	3.13	0.00302 *
	LE	Study	23	1.90	2.01	0.34	1.68	2.12	1.06	2.65	
I–V	RE	Control	15	4.5	4.88	0.71	4.45	5.24	2.90	5.82	0.08562
	RE	Study	23	4.47	4.50	0.41	4.32	4.79	3.66	5.11	
	LE	Control	15	4.39	4.55	0.52	4.23	4.78	2.98	4.97	0.00819 *
	LE	Study	23	4.95	4.92	0.60	4.60	5.38	3.67	5.87	

Key: Q1, first quartile; Q3, third quartile; N, number of participants; RE, right ear; LE, left ear; bold numbers, *p* ≤ 0.05 *.

**Table 4:** Results of amplitude (µV) of waves I and V and the V/I amplitude ratio in the Auditory Brainstem Response, considering the study group and the control group.

	Ear	Group	*N*	Mean	Median	SD	Q1	Q3	Min	Max	*p*-Value
Amplitude (µV)											
I	RE	Control	15	0.18	0.19	0.06	0.15	0.22	0.04	0.27	0.80572
	RE	Study	23	0.20	0.18	0.11	0.12	0.3	0.04	0.45	
	LE	Control	15	0.19	0.2	0.06	0.15	0.23	0.08	0.28	0.40502
	LE	Study	23	0.18	0.17	0.11	0.13	0.2	0.07	0.64	
V	RE	Control	15	0.28	0.25	0.08	0.24	0.29	0.20	0.52	0.22629
	RE	Study	23	0.30	0.27	0.15	0.18	0.35	0.12	0.67	
	LE	Control	15	0.32	0.27	0.13	0.22	0.45	0.15	0.53	0.43822
	LE	Study	23	0.26	0.25	0.09	0.19	0.34	0.13	0.39	
V/I ratio											
	RE	Control	15	1.76	1.50	0.97	1.21	1.89	1.05	5.00	0.43860
	RE	Study	23	1.80	1.80	0.90	1.33	2.13	0.40	4.75	
	LE	Control	15	1.71	1.72	0.51	1.21	2.07	1.00	2.63	0.76247
	LE	Study	23	1.57	1.63	0.48	1.20	1.92	0.56	2.29	

Key: Q1, first quartile; Q3, third quartile; N, number of participants; RE, right ear; LE, left ear.

## Data Availability

The raw data supporting the conclusions of this article will be made available by the authors on request due to privacy concerns.
